# Plate-Based High-Throughput Fluorescence Assay for Assessing Enveloped Virus Integrity

**DOI:** 10.1021/acs.biomac.4c00358

**Published:** 2024-07-23

**Authors:** Shannan-Leigh Macleod, Elana H. Super, Lauren J. Batt, Eleanor Yates, Samuel T. Jones

**Affiliations:** † Department of Materials and Henry Royce Institute, University of Manchester, Manchester M13 9PL, UK; ‡ School of Chemistry, University of Birmingham, Edgbaston, Birmingham, B15 2TT, UK

## Abstract

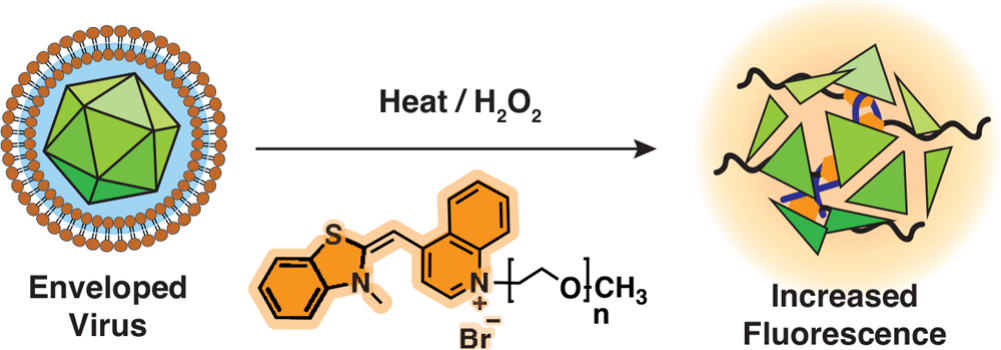

Viruses are a considerable threat to global health and place major burdens on economies worldwide. Manufactured viruses are also being widely used as delivery agents to treat (gene therapies) or prevent diseases (vaccines). Therefore, it is vital to study and fully understand the infectious state of viruses. Current techniques used to study viruses are often slow or nonexistent, making the development of new techniques of paramount importance. Here we present a high-throughput and robust, cell-free plate-based assay (FAIRY: Fluorescence Assay for vIRal IntegritY), capable of differentiating intact from nonintact enveloped viruses, i.e, infectious from noninfectious. Using a thiazole orange-terminated polymer, a 99% increase in fluorescence was observed between treated (heat or virucide) and nontreated. The FAIRY assay allowed for the rapid determination of the infectivity of a range of enveloped viruses, highlighting its potential as a valuable tool for the study of viruses and interventions against them.

## Introduction

Viruses account for approximately two-thirds of all known human pathogens^1^ and can place a significant burden on global healthcare systems and economies.^2,3^ They are also becoming widely used as delivery agents, in vaccines^4,5^ and in gene therapies.^6−8^ It is imperative to thoroughly study viruses and better understand infectious viral life cycles, yet current techniques are often slow or, in many instances, are nonexistent. Understanding the infectious state (i.e., infectious vs noninfectious) of a virus at each stage of its life cycle is critical. Such information feeds directly into the study of viruses and interventions against them (disinfectants, antiviral molecules, and surfaces), and into the development of virus-based gene therapies and vaccines.

A wide range of techniques to study viruses in a research setting exists, including: polymerase chain reaction (PCR), infectivity assays, flow virometry (FVM), transmission electron microscopy (TEM), enzyme-linked immunosorbent assays (ELISA), and high-throughput sequencing techniques,^9,10^ yet they are all unable to rapidly determine the infectious state of a virus. Of these, infectivity assays and PCR are commonly used to assess the presence of viruses. Infectivity assays, using cultured cells, are the gold standard for determining viral infectivity.^9^ However, they can be time-consuming and require skilled professionals to perform them.^11^ Furthermore, such studies rely on a cultivatable cell line, which is not available for all viruses e.g. human norovirus (HuNoV).^12^ This is concerning, as the next zoonotic or pandemic virus may lack cultivatable cell lines, which would significantly affect the development of vaccines and other interventions.

PCR can be used to quantify the amount of DNA or RNA in a sample, which is associated to the number of virions present.^9^ However, quantifying viral titer does not imply the presence of infectious virions.^13−16^ Attempts to combine quantitative PCR (qPCR) with intercalating azo-dyes have been explored to determine viral capsid integrity. Propidium monoazide (PMA) and ethidium monoazide (EMA) (or their derivatives PMAxx and PEMAX) are used to covalently bind the genetic material of compromised virions, preventing the subsequent nucleic acid amplification by qPCR.^17−21^ Hence, any signal observed would represent intact virions. However, results are not consistent, even when the same azo dye is used.^20,22^

Therefore, other techniques have been investigated with the hopes of overcoming the limitations of both PCR and cell-based studies, particularly fluorescence plate-based assays. Utilizing fluorophores, Walter et al., developed the plate-based thermal release assay (termed PaSTRy) to monitor viral stability. This assay monitors fluorescence changes of a range of small molecule nucleic acid^23−25^ and protein^23^ fluorophores upon heating. To date, PaSTRy has only been used to study the stability of picornaviruses^24,25^ and requires a wide range of fluorophores. To the best of our knowledge, there is currently no assay capable of identifying whether a sample contains intact or nonintact virions (i.e., infectious vs noninfectious), without slow cultivation in cells.

**Scheme 1:**
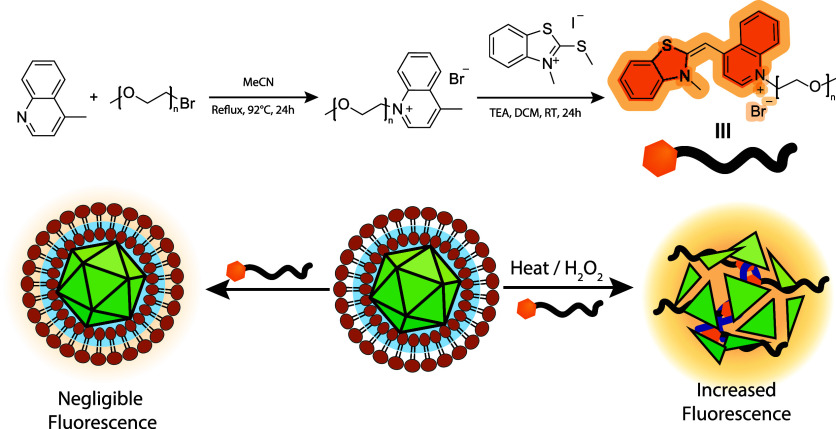
(A) Synthetic route to produce TO-PEG. (B) Schematic overview of the FAIRY assay showing the expected responses to intact and nonintact viral capsids.

Here, we report on a high-throughput and robust, cell-free assay that is capable of differentiating intact and nonintact enveloped viruses. It indirectly provides information regarding the infectivity of viral samples, i.e, intact capsid are infectious, while disrupted (or nonintact) capsids are noninfectious. To achieve this, we developed an assay that utilizes the size of a dye–polymer conjugate to halt premature penetration of intact virions, leading to a “turn-on” response only upon virion disruption. This unique fluorophore is utilized to determine the infectivity of herpes simplex virus-2 (HSV-2), cytomegalovirus (CMV) and respiratory syncytial virus (RSV) viral samples, under a range of conditions, in minutes (Scheme 1). This technique allows the infectious state of viral samples to be determined in minutes. Such a technique will enable rapid quality control during the manufacture of viral vectors for gene therapies and vaccines as well as for viral integrity related studies such as development and assessment of disinfectants and antiviral surfaces and screening new and existing antivirals.

## Experimental Section

### Materials

Reagents were purchased from Sigma-Aldrich and ThermoFisher Scientific unless otherwise stated. ^1^H NMR (nuclear magnetic resonance) (400 MHz) spectra were recorded using a Bruker Avance III. Chemical shifts were recorded in parts per million (ppm) (δ) using MeOH and DMSO as an internal reference set to δ 3.31 and δ 2.50 ppm. GPC measurements were carried out on an Agilent 1260 Infinity pump injection module, equipped with an Agilent 1260 Infinity II refractive index detector, variable wavelength detector, and three Phenomenex Phenogel columns connected in series, namely Phenogel 5u 500A (300 × 7.8 mm), Phenogel 5u 10E4A (300 × 7.8 mm) and Phenogel 5u 10E6A (300 × 7.8 mm), with a GPC eluent containing 100% triethylamine (THF). Calibration with a series of polystyrene sulfonate (PSS) was conducted prior to the measurement. All samples were filtered through 0.2 μm PTFE filters before injection. 2D DOSY (diffusion-ordered spectroscopy) (400 MHz) spectra were recorded by using a Bruker Avance III equipped with a BBO cryoprobe. A standard pulsed-field gradient DOSY sequence (Bruker “zg30” sequence) was used for data acquisition. Each DOSY spectrum was acquired with 64 scans, a relaxation delay of 1 s, and a spectral width of 30 ppm. Data was acquired using TopSpin version 3.6 in automation. MALDI-ToF measurements were carried out using a sDHB (10 mg/mL in 70% ACN and 0.1% TFA) matrix. The TO-PEG and PEG samples were dissolved in milli-q water. A 1:1 ratio (sample:matrix), at a volume of 1.5 μL, the mixture was air-dried on the steel target. MALDI-TOF mass spectra were acquired using the Bruker Rapiflex MALDI mass spectromety. The spectra were acquired using the average of 20,000 laser shots. Calibration was conducted prior to the measurements using a PEG 1,000 mixture. Fluorescent studies were performed using a black 96-well flat bottom plate with the lid on an Envision multimode monochromator plate reader by PerkinElmer Inc., (V1.13.3009.1394) using the following parameters: excitation and emission parameters were set at λ_*max*_ = 510 nm and λ_*max*_ = 533 nm, respectively; with the fluorescent cycle measurement set at a flash number of 100 with the photomultiplier tube detector being set at 750, unless otherwise stated.

### Synthesis of TO-PEG

#### Synthesis of Bromo-poly(ethylene) Glycol (PEG-Br) (SI Scheme 1)

PEG, average *M*_*n*_ 5,000 g/mol (18.0 g, 3.60 mmol, 3.0 equiv) was added to a three-neck flask. The reaction mixture was then left to stir at 100 °C until sufficiently melted. Potassium bromide (PBr_3_) (4.43 g, 1.20 mmol, 1.2 equiv) was then added dropwise to the melted PEG. The reaction mixture was stirred for 18 h at 100 °C. The reaction was then quenched with water (100 mL) and washed with dichloromethane (DCM), 0.1 M sodium thiosulfate solution, and saturated brine. The organic phase was isolated, dried over magnesium sulfate and filtered before the removal of the solvent under reduced pressure to yield an off-white solid (9.91 g, 44.7% yield). ^1^H NMR (400 MHz, DMSO-*d*_6_) (SI Figure 1) δ = 3.72 (m, H_*n*_); 3.24 (s, 3H) ppm.

#### Synthesis of 1-Poly(ethylene)-4-quinolinium Bromide (MQ-PEG) (SI Scheme 2)

PEG-Br (6.01 g, 1.20 mmol, 1.0 equiv) and 4-methylquinoline (MQ) (0.258 g, 1.80 mmol, 1.5 equiv) were added to a round-bottom flask and suspended in acetonitrile (20 mL). The reaction mixture was left to stir under reflux conditions for 24 h at 92 °C. The resulting suspension was dried under reduced pressure to yield a white solid. ^1^H NMR (400 MHz, MeOD-*d*_4_) (SI Figure 2) δ = 8.72 (d, 1H); 8.17 ppm (d, 1H); 8.02 (d, 1H); 7.79 (t, 1H); 7.67 (t, 1H); 7.43 (d, 1H); 3.72 (m, H_*n*_); 3.36 (s, 3H); 2.78 (s, 3H) ppm.

#### Synthesis of 3-Methyl-2-(methylthio)benzo[d]thiazol-3-ium Iodide (BC) (SI Scheme 3)

2-(Methylthio)benzothiazole (5.13 g, 28.29 mmol, 1.0 equiv) and iodomethane (8.03 g, 56.58 mmol, 2.0 equiv) were added to a round-bottom flask and degassed under inert atmosphere for 30 min. The reaction mixture was then left to stir for 4 h at 50 °C. The resulting white solid was dissolved in methanol and precipitated in diethyl ether (Et_2_O). The precipitate was collected via centrifugation, washed with Et_2_O (3 × 20 mL), and dried under reduced pressure to yield a white solid (1.05 g, 11.5%). ^1^H NMR (400 MHz, DMSO-*d*_6_) (SI Figure 3) δ = 8.42 (d, 1H); 8.18 (d, 1H); 7.84 (t, 1H); 7.72 (t, 1H); 4.11 (s, 3H); 3.36 (s, 3H) ppm.

#### Synthesis of TO-PEG, *M*_*n*_ = 5,000 g/mol (SI Scheme 4)

To the same round-bottomed flask in which MQ-PEG was synthesized, BC (0.62 g, 1.92 mmol, 1.6 equiv) was added and suspended in ethanol (10 mL) and DCM (2 mL). Triethylamine (0.321 mL, 2.30 mmol, 1.2 equiv) was added dropwise to the suspension, resulting in a dark red color change. The reaction mixture was then stirred overnight at room temperature. The resulting suspension was dried under reduced pressure to give a red solid. The resultant red solid was then purified by dialysis (1000 MWCO) (Spectrum Laboratories) against water for 5 days (3 water changes/day). The suspension was dried under reduced pressure to yield a red solid (5.55 g, 85.1% yield). ^1^H NMR (400 MHz, MeOD-*d*_4_) (SI Figure 4) δ = 8.74 (d, 1H); 8.50 ppm (d, 1H); 8.17 (d, H1); 7.95 (d, 1H); 7.79 (d, 1H); 7.73 (t, 1H); 7.65 (t, 1H); 7.53 (t, 1H); 7.46 (t, 1H); 7.27 (d, 1H); 5.95 (s, 2H); 4.07 (s, 3H); 3.64 (m, H_*n*_); 3.36 (s, 3H) ppm.

#### Synthesis of 2-Methoxyethyl 4-methylbenzenesulfonate Poly(ethylene) (PEG-OTs) (SI Scheme 5)

Tosyl chloride (TsCl) (Acros Organics) (0.253 g, 1.33 mmol, 1.2 equiv), PEG with average *M*_*n*_ 750 (0.83 g, 1.11 mmol, 1.0 equiv), and potassium hydroxide (KOH) (0.248 g, 4.43 mmol, 4 equiv) were added to a round-bottom flask and cooled in an ice bath. The reactants were dissolved in cool dry DCM (4 mL), under nitrogen conditions The reaction mixture was then left to stir for 3 h at room temperature. The reaction was then quenched with water (100 mL), centrifuged and filtered to remove excess KOH, and precipitated in Et_2_O to yield a white solid.

#### Synthesis of 1-Poly(ethylene)-4-quinolinium OTs (MQ-PEG-OTs) (SI Scheme 6)

PEG-OTs (0.0434 g, 0.048 mmol, 1 equiv) and MQ (9.51 μL, 0.072 mmol, 1.5 equiv) were added to a round-bottom flask and suspended in ACN (2 mL). The reaction mixture was left to stir under reflux conditions for 24 h at 92 °C. The resulting suspension was dried under reduced pressure yielding a white solid.

#### Synthesis of TO-PEG, *M*_*n*_ = 750 g/mol (SI Scheme 7)

To the same round-bottomed flask in which MQ-PEG-OTs was synthesized, BC (0.0372 g, 1.15 mmol, 1.6 equiv) was added and suspended in ethanol (1 mL) and DCM (0.2 mL). Triethylamine (12.04 μL, 0.086 mmol, 1.2 equiv) was added dropwise to the suspension, resulting in a dark red color change. The reaction mixture was then left to stir overnight at room temperature. The resulting suspension was dried under reduced pressure to yield a red solid. The resultant red solid was then purified by dialysis (100–500 MWCO) against water for 5 days (3 water changes/day). The suspension was dried under reduced pressure to yield a red solid. ^1^H NMR (400 MHz, MeOD-*d*_4_) (SI Figure 6) δ = 8.74 (d, 1H); 8.48 ppm (d, 1H); 8.16 (d, H1); 7.99 (d, 1H); 7.82 (t, 1H); 7.64 (t, 1H); 7.53 (t, 1H); 7.47 (t, 1H); 6.99 (s, 2H); 4.05 (s, 3H); 3.64 (m, H_*n*_); 3.36 (s, 3H) ppm.

### FAIRY Assay Development

#### Cell Culture

All cell culture was performed using aseptic techniques in a class II microbiological safety cabinet. Vero cells (ATCC (CCL-81)) were kindly donated by the University of Manchester, School of Medical Sciences (Prof. Pamela Vallely). They were maintained in Dulbecco’s Modified Eagle Medium (DMEM) modified with high glucose, phenol red (PR), supplemented with 1% penicillin/streptomycin (P/S) and 10% fetal bovine serum (FBS), unless otherwise stated. Vero cells were maintained at 37 °C in 5% CO_2_.

#### Viruses

Herpes Simplex Virus serotype two (HSV-2), Respiratory Syncytial Virus (RSV), and Cytomegalovirus (CMV) samples were originally isolated, verified, and kindly donated by the University of Manchester School of Medical Sciences (Professor Pamela Vallely). Additional viral stocks were expanded in-house on Vero cells using PR-free High glucose DMEM supplemented with 1% P/S and stored at −80 °C.

#### Wavelength Spectra of TO-PEG

To determine the wavelength spectra of TO-PEG, 50 μL of TO-PEG (0.5 mM) and 50 μL of extracted HSV-2 viral DNA (15 ng/μL) were added to the 96-well flat bottom plate and incubated for 10 min in the dark. The viral DNA was extracted using a Purelink viral RNA/DNA Mini Kit (Invitrogen), following the manufacturers guidance. The Envision multimode monochromator plate reader excitation and emission wavelengths parameters were set at 450 nm -520 and 518 nm -650 nm, respectively.

#### Fluorescent Properties of TO-PEG

To determine the intercalating properties of TO-PEG, the wavelength spectra, i.e., excitation and emission, parameters were set at λ_*max*_ = 510 nm and λ_*max*_ = 533 nm, respectively, using the fluorescent cycle measurement parameters mentioned previously. To a black 96-well flat bottom plate, 50 μL of TO-PEG (0.5 mM) and 50 μL of extracted HSV-2 viral DNA were added. For the control, RNAase-free water from the Purelink viral RNA/DNA Mini Kit was used instead of extracted HSV-2 viral DNA. All samples were incubated for 10 min in the dark.

#### Media Component Study

To a black 96-well flat bottom plate, 50 μL of TO-PEG (0.5 mM) and 50 μL of high glucose DMEM (1% P/S) (hereafter referred to as DMEM) containing phenol red (PR) and supplemented with 10% FBS were added. For the control, 50 μL of TO-PEG (0.5 mM) and 50 μL of milli-Q water were added. The samples were incubated for 10 min in the dark. Hereafter, the same procedure was used for PR-free DMEM supplemented with 10% FBS and PR-free DMEM (with no FBS supplemented). Using the Envision plate reader and the defined parameters, the fluorescence of TO-PEG was measured.

#### Capsid Destruction Using Heating

The first thawed HSV-2 sample was aliquoted into 6 eppendorf tubes. One eppendorf tube was placed on ice to maintain the integrity of the virions (hereafter referred to as nonheated). The other eppendorf tubes were heated accordingly, using a heat block, set at 50 °C, 70 °C, 80 °C, 90 and 100 °C, for 10 min. After heating, the eppendorf tubes were cooled for 5 min at room temperature. For the control, PR-free DMEM media (0% FBS) underwent the same treatment as the HSV-2 nonheated and heated samples. Hereafter, a titration assay, qPCR and the plate-based TO-PEG assay was performed.

#### Titration Assay

The nonheated and heated samples were serially diluted in PR-free DMEM (0% FBS) into 3 eppendorf tubes (1:10 dilution). Dilutions were then added in duplicate to Vero cells seeded on clear flat-bottom 96-well plates and titrated down the plate (1:3 dilution). The plates were incubated to allow virus adsorption for 1 h at 37 °C. Hereafter, this mixture was gently removed from the cells and overlaid with a 3:7 ratio of methyl cellulose (1.5 wt % methyl cellulose (Sigma-Aldrich, M0512) in deionized water) and 2% FBS, 1% P/S DMEM (hereafter referred to as MTC medium) and incubated for 24 h at 37 °C. Following incubation, the cells were fixed and stained with 0.5% crystal violet (Sigma-Aldrich). Virus titer was determined by CPE using a standard light microscope (10× objective), and the subsequent pfu/mL was calculated and compared to the nonheated HSV-2 sample.

#### qPCR Amplification

After heating the HSV-2 samples at the various temperatures, the viral HSV-2 DNA was extracted (the ethanol and lysis steps in the protocol was omitted) using the Purelink viral RNA/DNA Mini Kit (Invitrogen), as previously described.^26^ The extracted viral DNA was quantified by qPCR using the PowerUp SYBR Green Master Mix (Applied BioSystems), as previously described.^26^

#### Plate-Based TO-PEG Assay

To a black 96-well flat bottom plate was added 50 μL of TO-PEG (0.5 mM) and 50 μL of first thawed HSV-2 sample (nonheated) added. For the control, nonheated PR-free DMEM media (0% FBS) was used. Samples were incubated in the dark for 10 min and the fluorescence measured using the defined parameters (λ_*Ex*_ = 510 nm and λ_*Em*_ = 533 nm). The same procedure was used for the heated HSV-2 samples, and the other virus studied i.e. RSV and CMV.

#### Known Virucides

The known antiviral chemicals (virucides), 2% hydrogen peroxide (H_2_O_2_) (Sigma-Aldrich), 50% ethanol (EtOH) (Sigma-Aldrich), and 50% isopropanol (IPA) (Sigma-Aldrich), were sterilized using a 0.22 μm filter. These virucides were added at a 1:1 ratio with HSV-2 and incubated for 1 h at 37 °C. To a black 96-well flat bottom plate, 50 μL of TO-PEG (0.5 mM) and 50 μL of the chemically treated samples (and nontreated samples) were added. Samples were incubated in the dark for 10 min and the fluorescence measured using the defined parameters (λ_*Ex*_ = 510 nm and λ_*Em*_ = 533 nm).

#### Statistical Analysis

Data were presented as mean ± SD (n = 3), unless otherwise stated. Statistical analyses were performed using GraphPad Prism version 9.1, using one-way ANOVA, unless otherwise stated in the figure legends. P-values considered statistically significant are represented with *p < 0.02, **p < 0.01 and ***p < 0.001.

## Results and Discussion

Cyanine-based dyes are a highly versatile and widely used class of dyes that have been developed to function over a wide range of wavelengths.^27−29^ The asymmetric cyanine nucleic acid fluorophore, Thiazole Orange (TO), has gained much attention,^30^ particularly for its use in biosensors.^31−35^ In an aqueous solution, the quantum yield of TO is very low (Φ = 0.0002) due to the free rotation of the benzothiazole and quinoline heterocycles, meaning that fluorescence is negligible. Meanwhile, when bound to nucleic acids this free rotation is restricted, resulting in a significant fluorescent yield increase of approximately 1,000-fold.^31^ However, in aqueous solutions, the hydrophobic, nonpolar, aromatic hydrocarbon rings leads to the formation of TO aggregates. This reduces the number of TO molecules available for nucleic acid association and consequently the resulting fluorescence is reduced.^36^ Additionally, small molecule fluorophores, including TO and SYBR Green II (another cyanine dye), have been shown to penetrate intact viral capsids, through transiently open pores during viral capsid “breathing”.^37−42^

In order to allow for the development of this fluorescence assay for vIRal IntegritY (FAIRY) plate-based assay, capable of distinguishing intact from nonintact viruses, we hypothesized that an increase in both the size and solubility of TO is necessary. Increasing the size of TO, would prevent the premature penetration of TO into intact virions during viral capsid “breathing”. Additionally, increasing the solubility of TO, would prevent the formation of TO aggregates, resulting in a greater proportion of TO molecules available for association with nucleic acids.

One of the simplest approaches to simultaneously control size and improve solubility was through the conjugation of TO to a hydrophilic polymer. Here, the water-soluble and biocompatible polymer polyethylene glycol (PEG) is attached to TO, producing a hydrophilic and, importantly, larger TO-polymer conjugate (termed TO-PEG) (SI Figures 1–4). TO-PEG was synthesized by first reacting 4-methylquinoline (MQ) with bromo-terminated PEG, which was subsequently reacted with 2-(methylthio)benzothiazole (BC). The presence of a peak at 5.95 ppm (annotated as “i” in Figure S4 SI) confirms that TO has been formed. The ratio of integrals between peak ’i’ and those for PEG confirm the successful synthesis of TO-PEG. The final product and all intermediates were fully characterized (see SI) before further experiments were conducted. PEG is commercially available in a wide range of molar masses and an understanding of the link between molar mass and hydrodynamic size in water, defined as radius of gyration, is well understood. A formula to calculate the radius of gyration, from molar mass, has also been developed: *R*_g_ = 0.02*M*^0.58^, where *R*_g_ = radius of gyration and *M* = molar mass.^43^ Viral capsid pores range in size from 0.6 to 2 nm.^38,44−48^ In order to ensure that TO-PEG is unable to access the genetic material of intact virions, a radius of gyration > 2 nm is required. A PEG with molar mass of 5,000 g/mol has a calculated radius of gyration of ∼2.8 nm.^43^ Hence, when attached to TO, the formed TO-PEG should be sufficiently large enough not to prematurely penetrate an intact capsid, even through transiently open pores.

**Figure 1:**
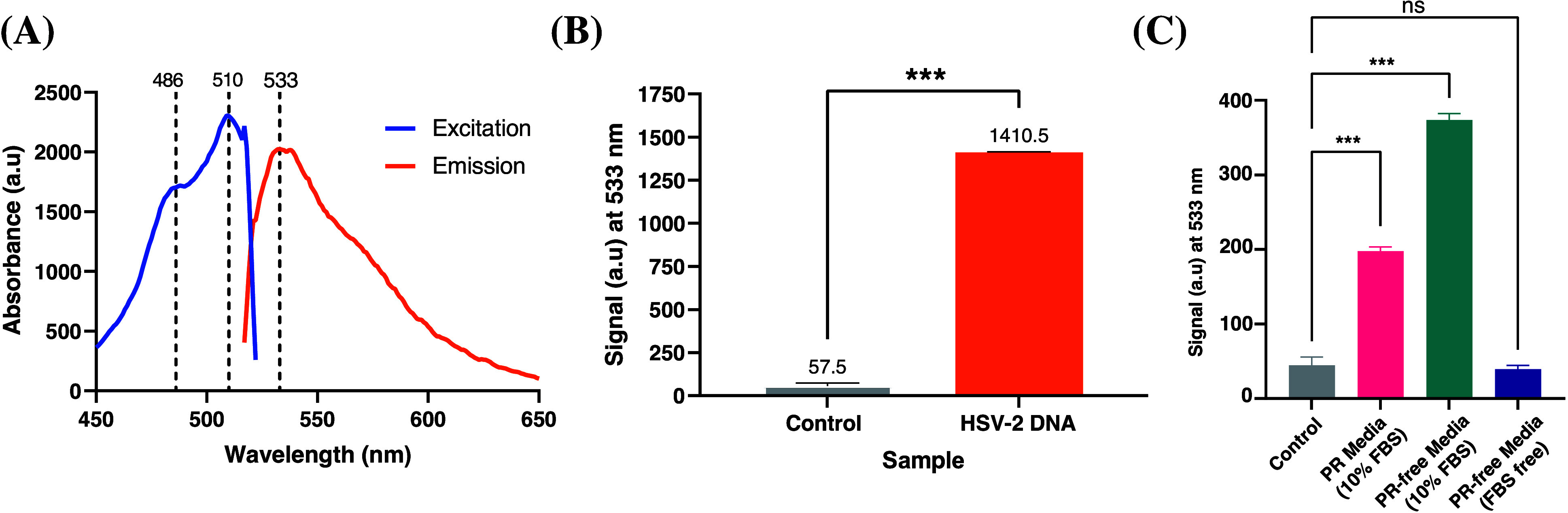
Fluorescent properties of TO-PEG. (A) Excitation and Emission spectra. (B) Fluorescence signal before and after mixing TO-PEG with extracted HSV-2 DNA. Statistical analyses were performed using unpaired, parametric *t* test (*** *p* < 0.001). (C) Investigating the effect on fluorescence of media components on TO-PEG. Key: PR = Phenol Red. Data are presented as mean ± SD. Statistical analyses were performed using one-way ANOVA (*** *p* < 0.001, ns = nonsignificant).

To verify that the fluorescent properties of TO had not been altered through attachment to a polymer, the excitation and emission of TO-PEG were investigated. TO absorbs and emits at λ_*ex*,*max*_ = 510 ± 5 nm and λ_*em*,*max*_ = 533 ± 5 nm, respectively^49^ (SI Figure 5), and as PEG is not itself fluorescent, a similar wavelength is expected for TO-PEG. Using a monochromatic plate reader and extracted HSV-2 viral DNA, it was confirmed that the absorbance and emission wavelengths of TO-PEG remained at λ_*ex*,*max*_ = 510 nm and λ_*em*,*max*_ = 533 nm, respectively (Figure 1A). Using these defined wavelengths, a 1,300-fold increase in fluorescence was observed for TO-PEG with HSV-2 DNA (15 ng/μL) vs no DNA (Figure 1B). This study confirms that the addition of PEG does not appear to inhibit the intercalation of TO to extracted viral DNA.

Before further studies with viruses, it was necessary to determine whether standard media components would interfere with the fluorescence of TO-PEG. Phenol red (PR), for example, which is a standard media ingredient, is known to interfere with fluorescent studies, despite emitting at λ_*max*_ = 570 nm.^50^ In order to determine which media components may affect the fluorescence of TO-PEG, a systematic study was undertaken in which media components were removed. Due to its universal use in cell culture, standard PR media supplemented with 10% fetal bovine serum (FBS) was investigated with 0.5 mM TO-PEG. A roughly 4-fold increase in fluorescence was observed when compared to TO-PEG in water (control) (Figure 1C). This fluorescence increase was unexpected due to the lack of nucleic acids. Therefore, some component in the media must be interacting with TO-PEG. Due to the previously reported potential for PR to impact fluorescent studies, PR-free media (supplemented with 10% FBS) was then analyzed with 0.5 mM TO-PEG. In this instance, a roughly 8-fold increase in fluorescence, compared to the control, was observed. The unexpected increase in fluorescence had not only remained but also without PR had increased further, indicating another media component must be having an effect on TO-PEG. As a media supplement, FBS can be easily removed or its concentration readily controlled. Therefore, PR- and FBS-free media was investigated. The resulting fluorescence had little to no change when compared to the control, indicating the negative impact both FBS and PR had on the fluorescent properties of TO-PEG. Therefore, in all subsequent studies, PR- and FBS-free media was used.

It was then necessary to investigate if the synthesized TO-PEG was sufficiently large to prevent premature penetration into intact viral capsids. For this study, the enveloped DNA virus, HSV-2 was used on account of its ease of study and propagation. Intact HSV-2 (virus that is used directly after the first thawing) should, assuming that TO-PEG is too large to access the encapsulated viral DNA, have minimal fluorescence. First thawed HSV-2 samples were used in order to reduce the amount of released viral DNA (i.e., background), which occurs due to repeated freeze–thaw cycles. In order to show that HSV-2 was intact, it was necessary to produce nonintact HSV-2 through other means, such as heating. Heating leads to the destruction of the viral capsid^23^ and should therefore allow TO-PEG access to the viral DNA, resulting in an increase in fluorescence. Before this FAIRY assay could be further developed, the optimal temperature for the HSV-2 capsid destruction needed to be determined.

Previous studies suggest HSV-2 has one of the most robust capsids, and consequently can withstand high temperatures of up to 75–80 °C before denaturation occurs.^51−53^ A thermal scan was then conducted, in which HSV-2 samples were heated to various temperatures, ranging from room temperature to 100 °C, for 10 min. Temperature induces not only capsid destruction but also structural changes to proteins including receptors. Viral capsids have receptor’s that have vital roles in the initial stages of the viral replication cycle.^54,55^ An increase in temperature may disrupt the HSV-2 replication cycle, even before capsid denaturation occurs. Therefore, intact but defective virions may be present that are incapable of replicating. It was therefore necessary to investigate both defective and intact virions of the heated HSV-2 samples, i.e., noninfectious and infectious virions, before any fluorescent studies were undertaken.

**Figure 2:**
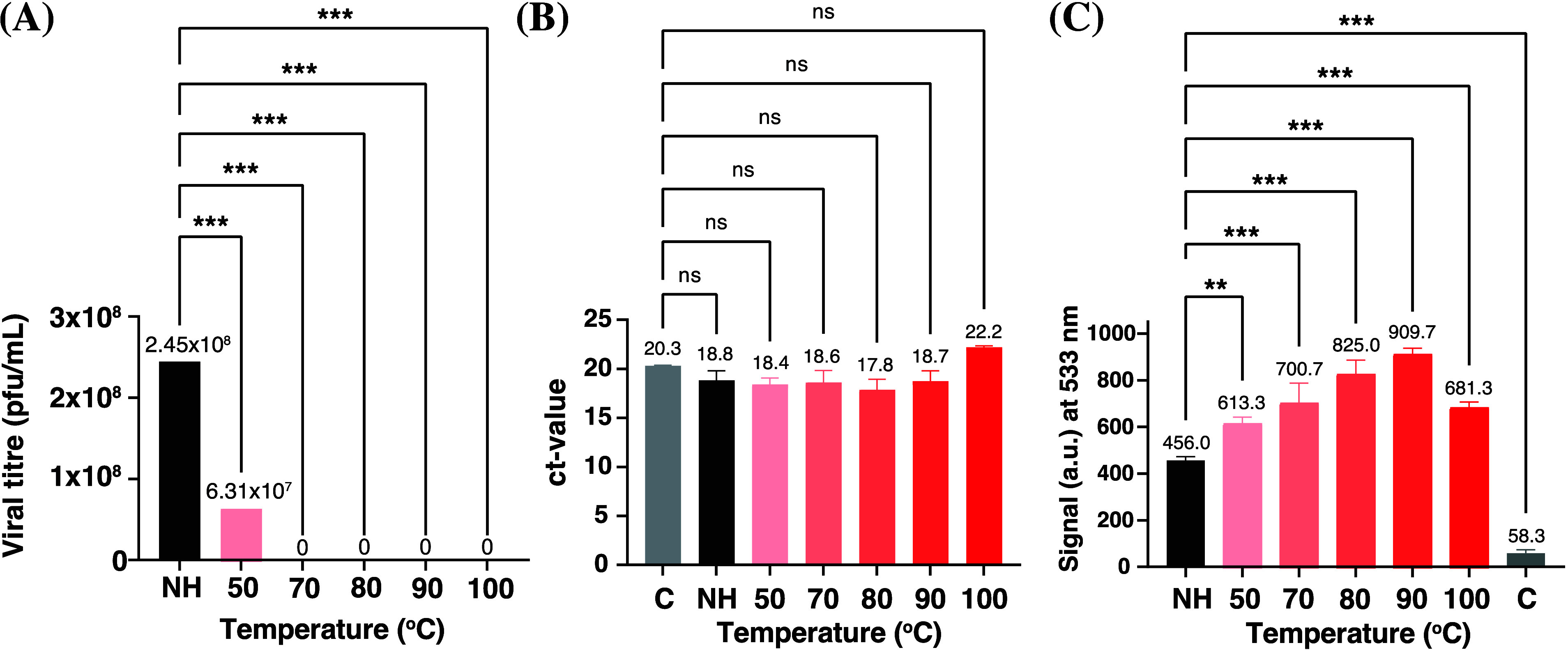
Assessing HSV-2 following heating over a range of temperatures (50–100 °C). (A) Quantifying infectious virions using a standard 96-well plate plaque-counting technique. (B) Quantifying the amount of HSV-2 DNA present in both nonheated and heated samples using qPCR. (C) Monitoring fluorescence, using TO-PEG, of HSV-2 after inducing capsid destruction through heating. Key: C = Control (media only), NH = Nonheated. Data are presented as means ± SD. Statistical analyses were performed using one-way ANOVA (**p < 0.01, ***p < 0.001, ns = nonsignificant).

Consequently, standard plaque counting in a 96-well plate was used to determine the plaque forming units per milliliter (PFU/mL) of each heated sample. The nonheated sample had a high viral titer (2.45 × 10^8^ PFU/mL). However, heating at 50 °C for 10 min resulted in a 4-fold decrease in viral titer, with no infectious virus being detected from 70 °C - 100 °C (Figure 2A). To ensure the differences in PFU of each heated sample was due to less infectious virions, it was necessary to quantify the total amount of HSV-2 DNA from infectious and noninfectious virions in each sample. This was determined using qPCR, as heating should not alter the quantity of HSV-2 DNA. The amount of HSV-2 DNA present in the samples was comparable to the HSV-2 reference genome (positive control) cycle threshold (cT) value of approximately 20 (Figure 2B). Confirming the amount of HSV-2 DNA in the nonheated and heated samples were consistent. This further shows that qPCR is incapable of discriminating between infectious and noninfectious virions. Hence, when infectious virions need to be quantified, qPCR can not be used. Although it has been confirmed that heating reduces HSV-2 infectivity, the optimal temperature at which the viral DNA is released and accessible to TO-PEG is vital to be able to apply this assay to the study of a range of virucidal compounds.

**Figure 3:**
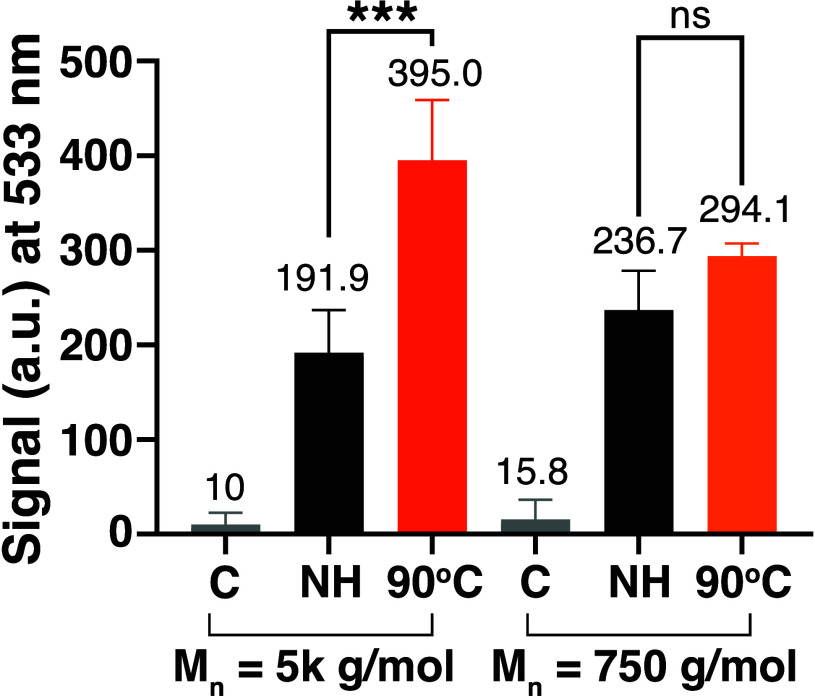
FAIRY assay comparison between molecular weights of TO-PEG, namely, 5K and 750 g/mol, with HSV-2 virions following capsid destruction using heat at 90 °C for 10 min. Key: C = Control, NH = Nonheated. Data are presented as means ± SD. Statistical analyses were performed using one-way ANOVA (***p < 0.001, ns = nonsignificant).

Standard quantification methods are unable to provide information regarding the infectivity of such heated samples. Therefore, over the same temperature range, the fluorescence of HSV-2, incubated with TO-PEG (0.5 mM), were investigated (λ_*ex*,*max*_ = 510 nm, λ_*em*,*max*_ = 533 nm) using a plate reader (Figure 2C). The optimal temperature for breaking of the viral capsid will be determined by the highest fluorescent signal. At every temperature, significant differences in fluorescence were observed between the heated samples and the nonheated sample, particularly at 80 and 90 °C (*P* < 0.001). The largest fluorescence increase was observed for samples heated at 90 °C for 10 min, suggesting that this is the optimal temperature for TO-PEG accessibility to viral HSV-2 DNA. It has previously been shown that DNA degrades at temperatures greater than 90 °C,^56,57^ providing an explanation for the decrease in fluorescence observed for HSV-2 heated at 100 °C. Nonetheless, with a 99% increase in fluorescence (Figure 3), it was confirmed that the FAIRY assay utilizing TO-PEG is capable of differentiating intact and nonintact HSV-2.

Previously we targeted a PEG radius of gyration of >2 nm in order to avoid premature penetration of viral capsids. In order to confirm this hypothesis, a smaller TO-PEG, with a molecular weight of 750 g/mol, was synthesized (SI Figure 6). According to the radius of gyration formula, a PEG polymer with a molar mass of 750 g/mol has a calculated radius of gyration of 0.9 nm. Therefore, in theory, during capsid “breathing”, this TO-conjugated 750 g/mol polymer has a high probability of prematurely penetrating the intact virion through the transiently open pores. Using the optimal capsid disruption temperature previously determined for HSV-2 of 90 °C for 10 min, followed by a 10 min incubation of the sample with TO-PEG-750 (0.5 mM), no significant difference was observed between the heated and nonheated samples (Figure 3). Showing that a radius of gyration of 0.9 nm did not prevent premature binding with the genomes of intact virions and was thus not sufficient to differentiate between intact and nonintact viruses.

**Figure 4:**
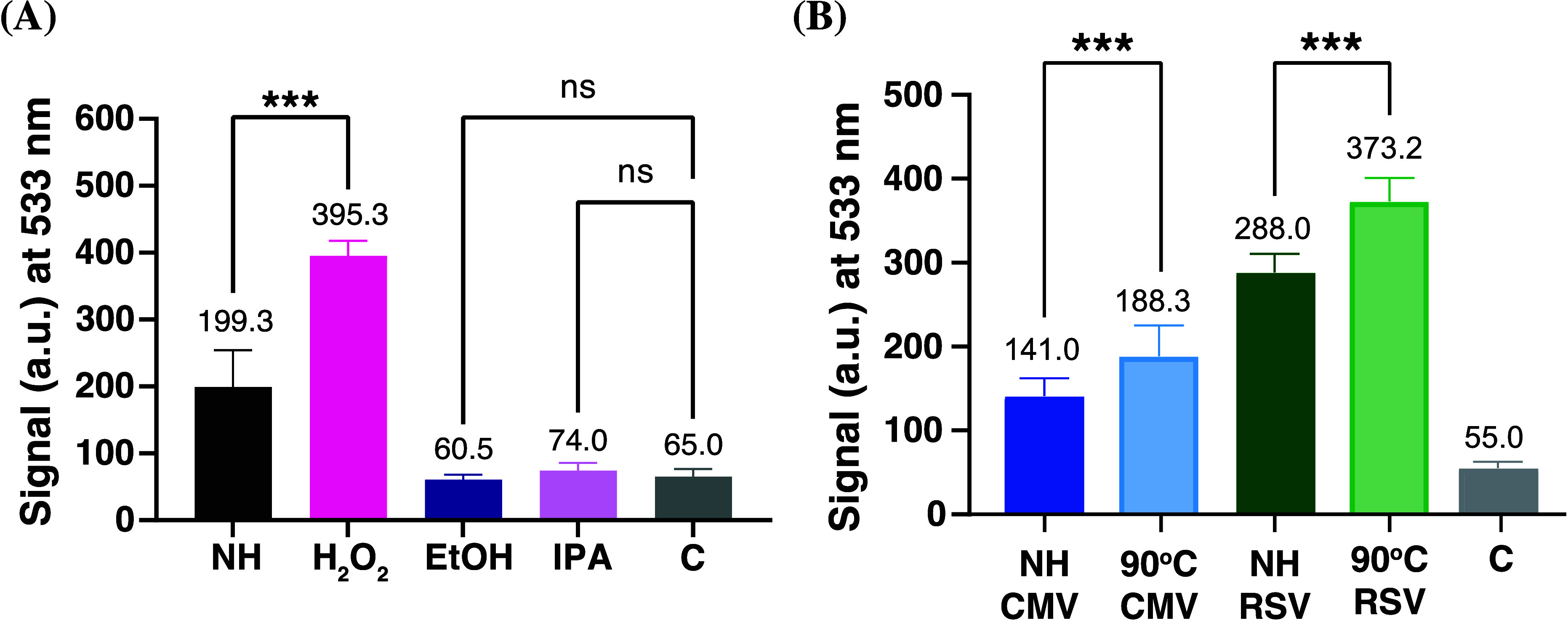
FAIRY assay used to monitor HSV-2 capsid destruction using (A) known virucides including 2% hydrogen peroxide (H_2_O_2_), 50% ethanol (EtOH), and 50% isopropanol (IPA) and (B) probing FAIRY assay robustness using different enveloped viruses, namely, CMV and RSV. Key: C = Control, NH = Nonheated. Data are presented as means ± SD. Statistical analyses were performed using one-way ANOVA (**p < 0.01, ***p < 0.001, ns = nonsignificant).

To further develop and demonstrate the robustness of the FAIRY assay, it was necessary to determine whether alternative capsid destruction methods (i.e., known virucides) could also be interrogated. It was vital to demonstrate that TO-PEG intercalation and therefore fluorescent properties were not altered due to the presence of these virucides. To do this, chemicals with broad-spectrum virucidal activity, including 2% hydrogen peroxide (H_2_O_2_), 50% Ethanol (EtOH) and 50% isopropanol (IPA),^58,59^ were incubated with HSV-2 for 1 h. Following this incubation, the treated and nontreated samples were incubated with TO-PEG (0.5 mM) for 10 min and the fluorescence investigated using a plate reader. Unexpectedly, for the EtOH and IPA samples, a low fluorescence signal was observed. Both of these alcohols are well-known virucides and so an increase in fluorescence was expected. In this instance, the low fluorescence signal is believed to be linked to the precipitation of genomic material or removal of the viral envelope (and so loss of infectivity)^60^ without breaking of the viral capsid. In some instances intact virions can also be noninfectious, particularly if capsid remained intact during the disruption of the envelope.^61^ Precipitation of the genomic material would mean that TO-PEG’s accessibility to the DNA would have been severely reduced (Figure 4A). On the other hand, a 99% fluorescence increase was observed between the nontreated and H_2_O_2_ treated samples, confirming the FAIRY assay has potential for the study of virucides that destroy viral capsids.

In order to investigate whether the FAIRY assay is more broadly applicable to other viruses, two further enveloped viruses were investigated. These each have different genomic materials (DNA vs RNA) allowing the applicability of the FAIRY assay to be probed. Here we used CMV, which is an enveloped DNA virus, and RSV, which is an enveloped RNA virus (Figure 4B). Heating was used as the capsid destruction method, with 90 °C for 10 min showing a significant reduction in infectious viral particles for both CMV (SI Figure 7) and RSV (SI Figure 8). Following capsid destruction through heating, the samples were incubated with TO-PEG (0.5 mM) for 10 min and the fluorescence was determined. An approximate 34% and 30% fluorescence increase was observed for CMV and RSV, respectively, further proving the robustness of the FAIRY assay for DNA and RNA enveloped viruses.

## Conclusion

Here, we report a high throughput, robust, cell-free TO-PEG plate-based FAIRY assay to study the infectivity of viral samples, a technique that has been shown to overcome some of the challenges of current techniques. Through the coupling of TO to a large water-soluble polymer (PEG), the overall size and water solubility of the dye was increased. It was shown that this increase in size prevented the premature penetration of the dye to intact viral capsids, exemplified initially using HSV-2. This was further confirmed using a lower molar mass PEG, whose radius of gyration was less than the capsid pore, which was unable to distinguish intact from nonintact virions. Utilizing the 5K TO-PEG, allows for the rapid differentiation between intact and nonintact (infectious vs noninfectious) enveloped viruses, in PR-free DMEM (0% FBS), in minutes. This FAIRY assay has demonstrated its superiority compared to other techniques and was shown to be probed by different capsid destruction methods, including heating and hydrogen peroxide. The robustness of the FAIRY assay was demonstrated through the use of further DNA and RNA enveloped viruses (CMV and RSV, respectively). This new assay has significant potential for the study of known viral destruction methods and novel virudical agents, which is crucial for combating the threat posed by viruses.

## Supporting Information Available

^1^H NMR, GPC and MALDI-ToF spectra for all compounds, synthetic schemes, wavelength spectra of commercial TO and titration assays for CMV and RSV (PDF)
