# Supplementary material for: Plate-Based High-Throughput Fluorescence Assay for Assessing Enveloped Virus Integrity
Source: Biomacromolecules. 2024 Jul 23;25(8):4925–33. doi: 10.1021/acs.biomac.4c00358 (PMC11323024; doi:10.1021/acs.biomac.4c00358)
Supplement: Supplementary file 1 — bm4c00358_si_001.pdf [file bm4c00358_si_001.pdf]

A plate-based high-throughput fluorescence assay for assessing enveloped virus integrity

Shannan-Leigh Macleod,† Elana H. Super,† Lauren J. Batt,† Eleanor Yates,‡ and Samuel T. Jones*,‡,†

†Department of Materials and Henry Royce Institute, University of Manchester, Manchester, M13 9PL, and ‡School of Chemistry, University of Birmingham, Edgbaston, Birmingham, B15 2TT

E-mail: s.t.jones.1@bham.ac.uk

Supporting Information

Supporting Information Available

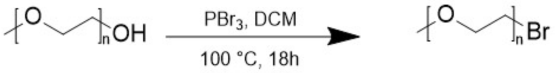

SI Scheme 1: Synthesis of bromo-poly(ethylene) glycol (PEG-Br)

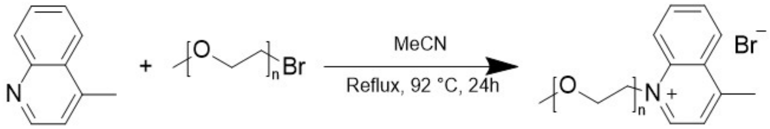

SI Scheme 2: Synthesis of 1-poly(ethylene)-4-quinolinium bromide (MQ-PEG)

*To whom correspondence should be addressed
†University of Manchester
‡University of Birmingham

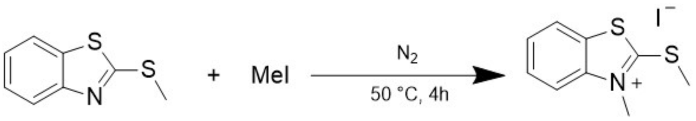

SI Scheme 3: Synthesis of 3-methyl-2-(methylthio)benzo[d]thiazol-3-ium iodide (BC)

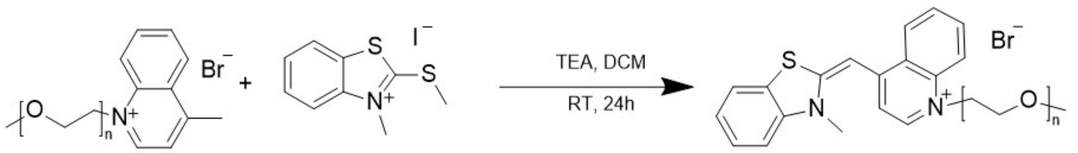

SI Scheme 4: Synthesis of **TO-PEG**, M_n = 5,000 g/mol.

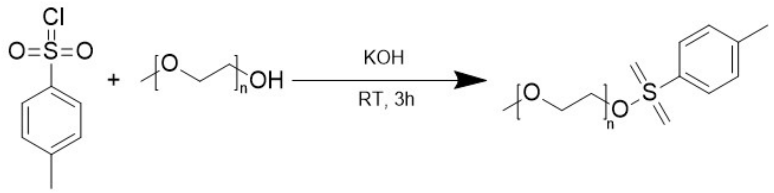

SI Scheme 5: Synthesis of 2-methoxyethyl 4-methylbenzenesulfonate poly(ethylene) (PEG-OTs)

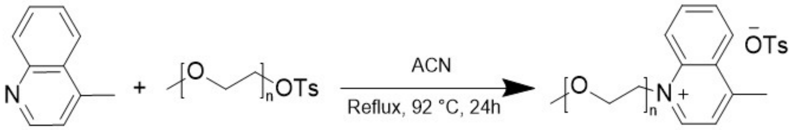

SI Scheme 6: Synthesis of 1-poly(ethylene)-4-quinolinium OTs (MQ-PEG-OTs)

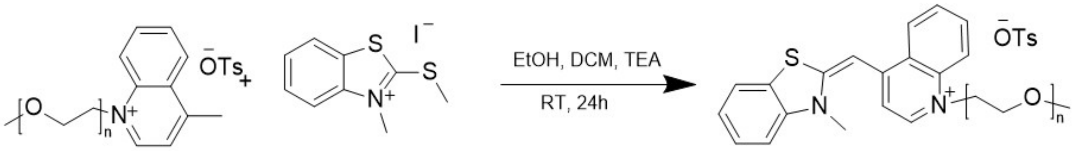

SI Scheme 7:Synthesis of TO-PEG, M_n = 750 g/mol.

‘

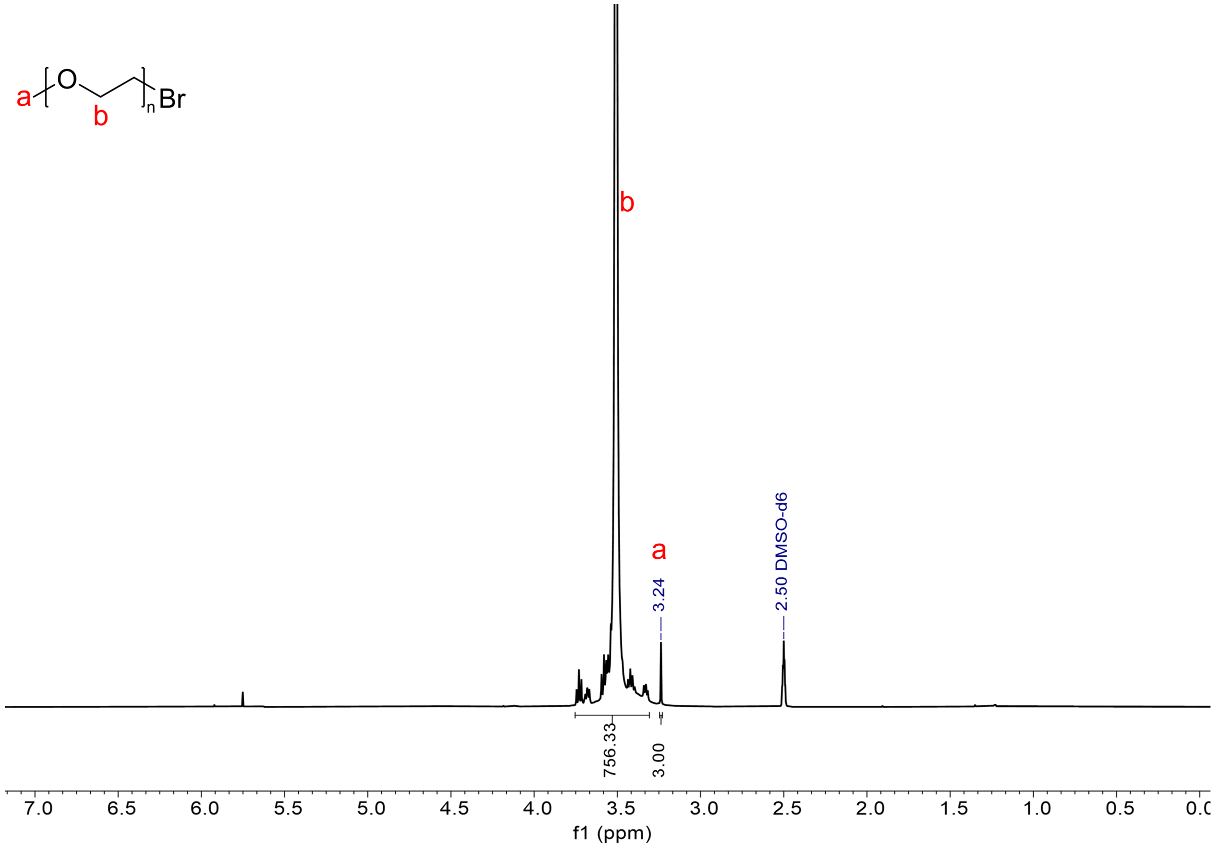

SI Figure 1: ^{1}H NMR spectrum of PEG-Br in DMSO-d6. The chemical structure of PEG-Br shows the assignment of the proton environments.

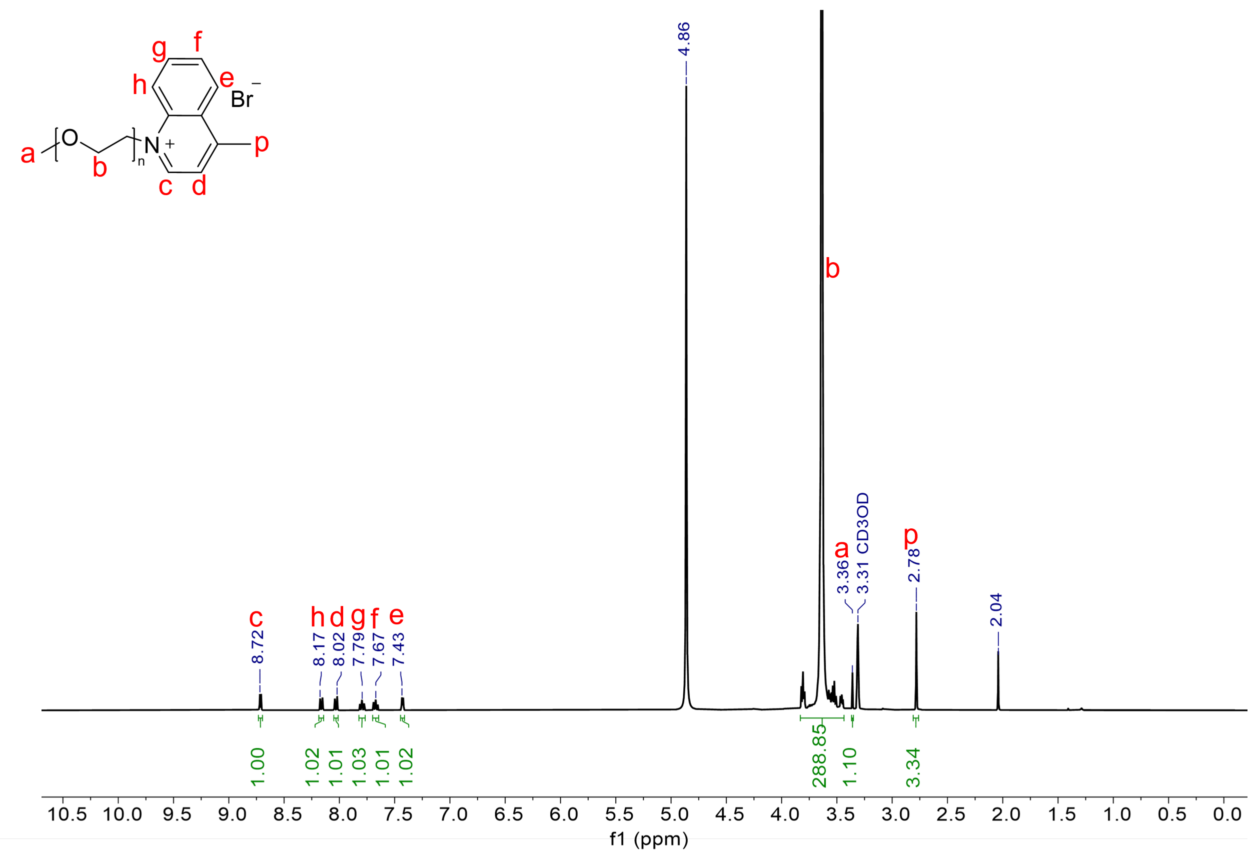

SI Figure 2: ^{1}H NMR spectrum of 1-poly(ethylene)-4-quinolinium bromide (MQ-PEG) in MeOD-d4. The chemical structure of MQ-PEG shows the assignment of the proton environments.

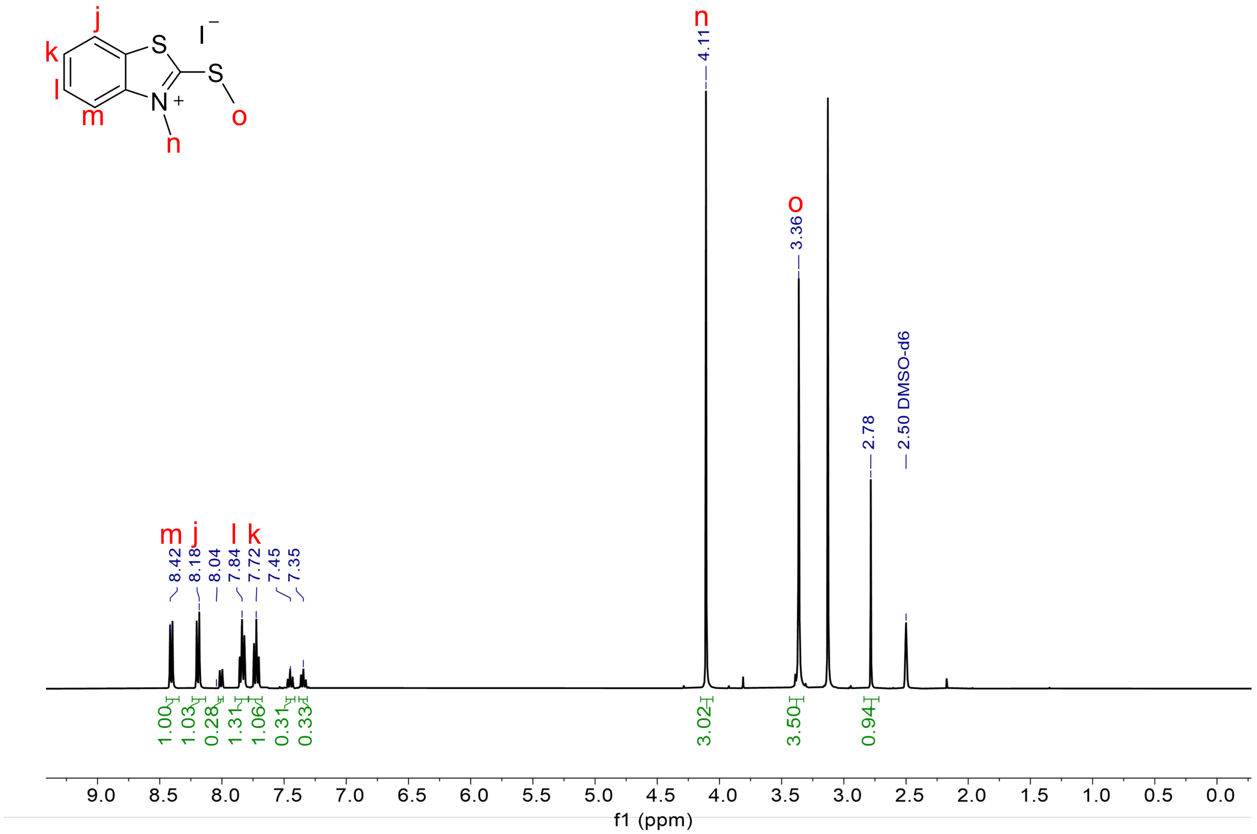

SI Figure 3: ^{1}H NMR spectrum of 3-methyl-2-(methylthio)benzo[d]thiazol-3-ium iodide (BC) in DMSO-d6. The chemical structure of BC shows the assignment of the proton environments.

(A)

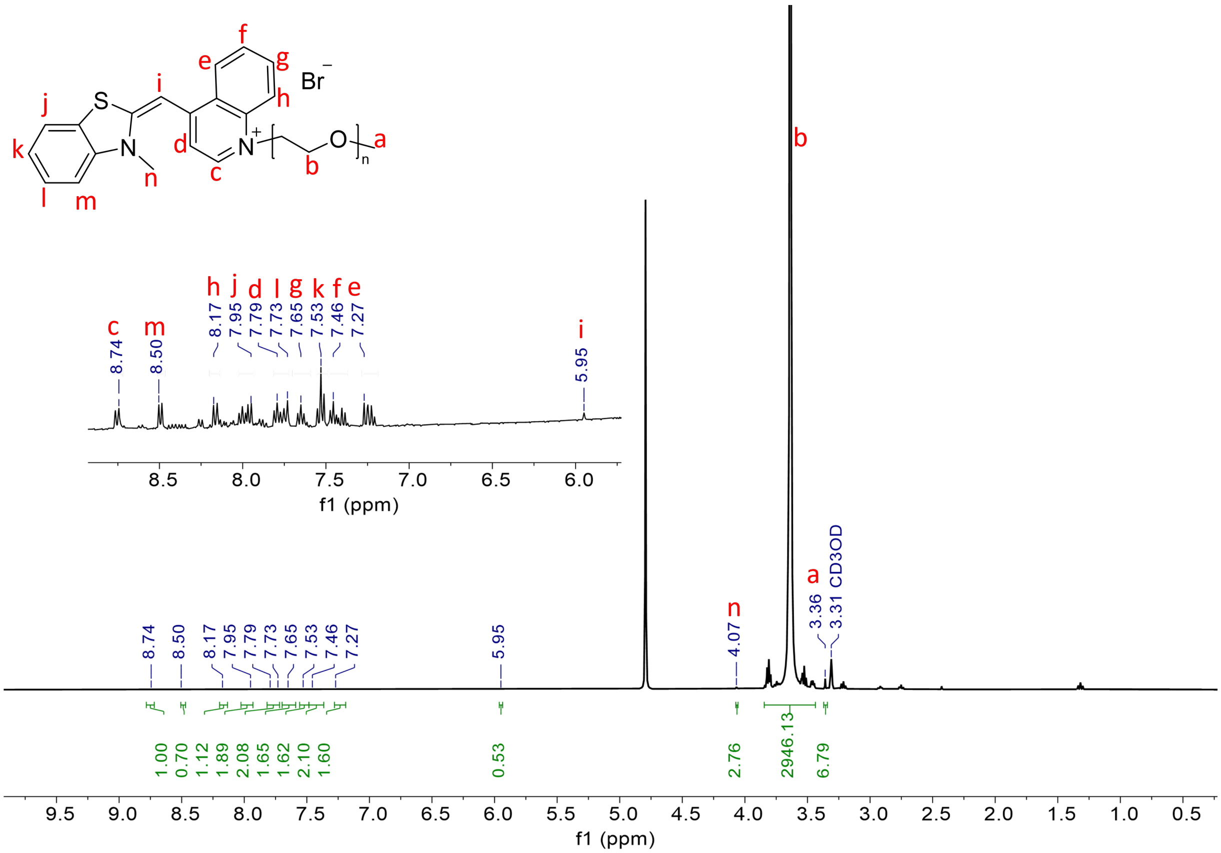
Br⁻
b
a
n
CD3OD
8.74
8.50
8.17
7.95
7.79
7.73
7.65
7.53
7.46
7.27
5.95
4.07
3.36
3.31
1.00
0.70
1.12
1.89
2.08
1.65
1.62
2.10
1.60
0.53
2.76
2946.13
6.79
f1 (ppm)

(B)

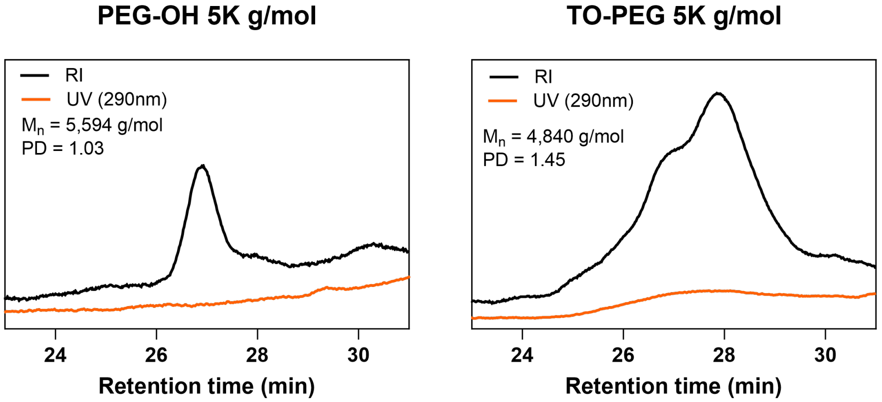
PEG-OH 5K g/mol
RI
UV (290nm)
Mn = 5,594 g/mol
PD = 1.03
Retention time (min)
TO-PEG 5K g/mol
RI
UV (290nm)
Mn = 4,840 g/mol
PD = 1.45
Retention time (min)

(C)

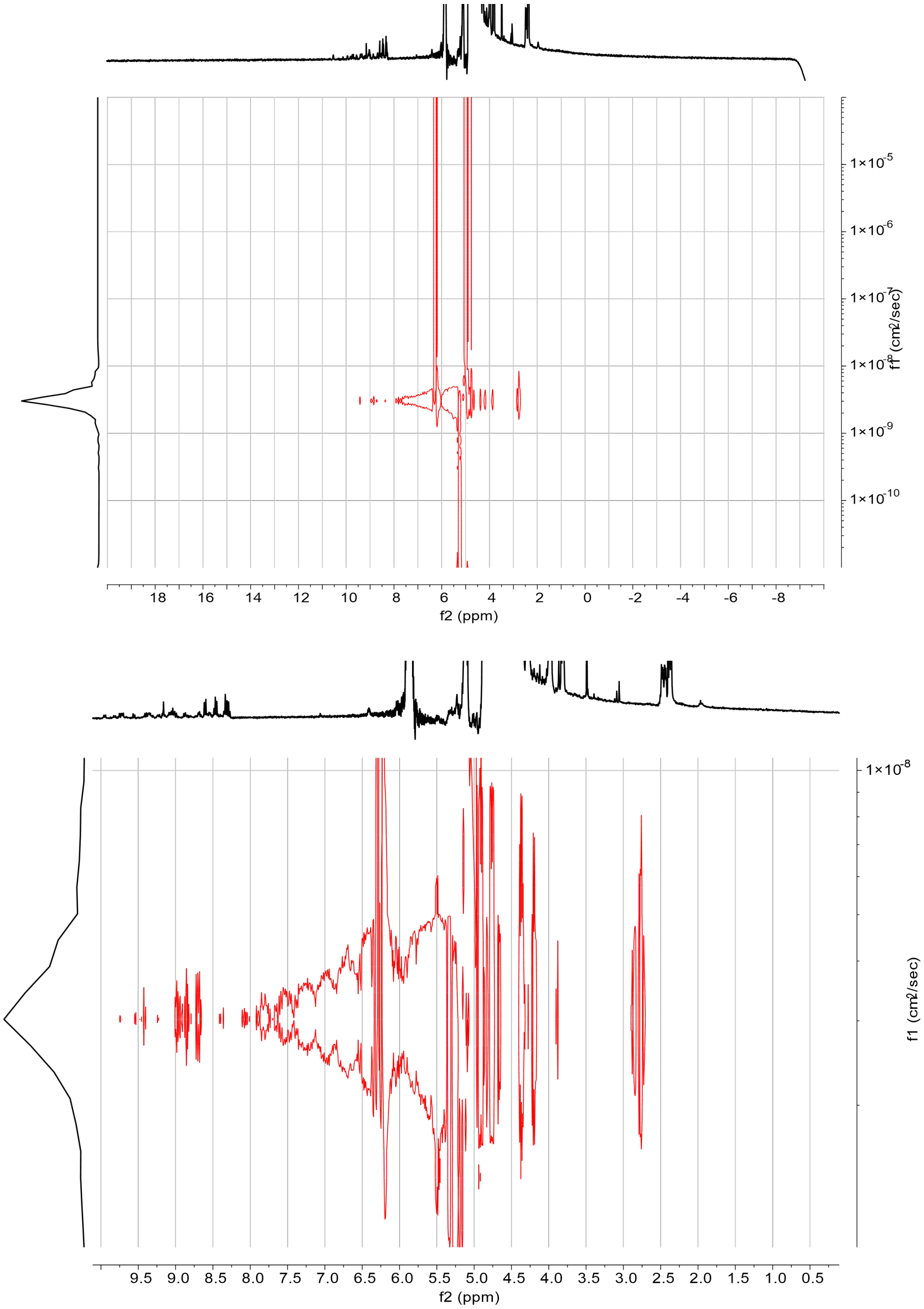

SI Figure 4: **TO-PEG 5K** characterisation. **(A)** ^{1}H NMR spectrum of **TO-PEG 5K** in MeOD-d4 with an inset of the aromatic environment peaks. The chemical structure of **TO-PEG** shows the assignment of the proton environments. The environment peak (labelled "i") shows the successful functionalisation of MQ-PEG to BC, resulting in the successful synthesis of **TO-PEG 5K**. **(B)** GPC spectrum of PEG-OH 5K (left) and **TO-PEG 5K** (right) with a refractive index and UV trace. The GPC results confirms **TO-PEG 5K** is of similar size to PEG OH, and that TO is successfully attached to PEG. **(C)** DOSY spectra showing **TO-PEG 5K** diffuses at the same rate, confirming the attachment of the molecules.

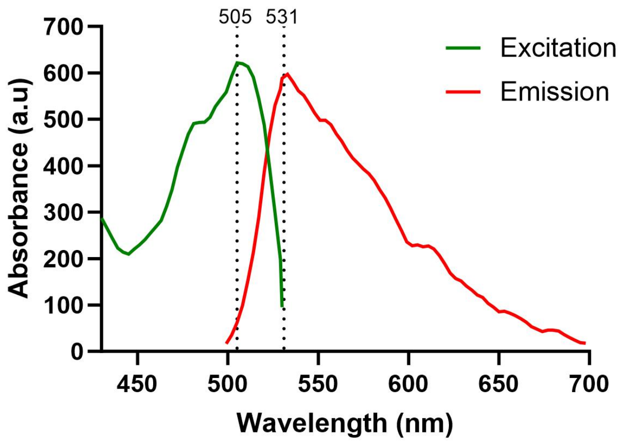

SI Figure 5: Excitation and Emission spectra of commercial Thiazole Orange (TO).

(A)

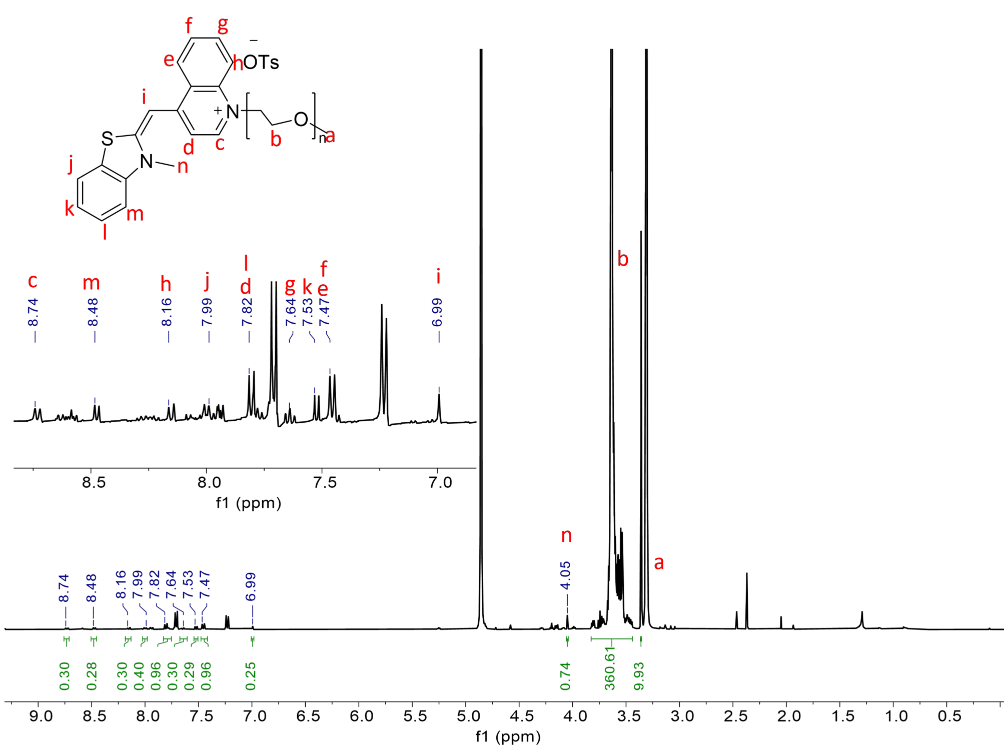

(B)

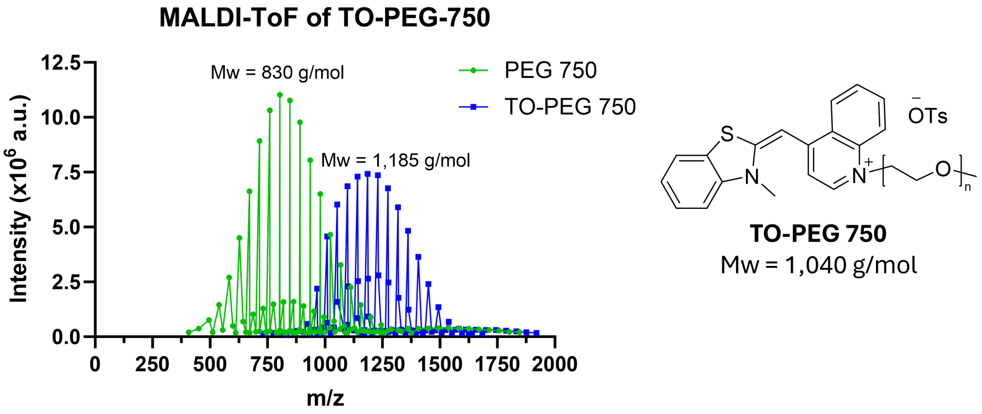

SI Figure 6: **TO-PEG 750** characterisation. **(A)** ^{1}H NMR spectrum of **TO-PEG 750** in MeOD-d4 with an inset of the aromatic environment peaks. The chemical structure of **TO-PEG** shows the assignment of the proton environments. The environment peak (labelled “i") shows the successful functionalisation of MQ-PEG-OTs to BC, resulting in the successful synthesis of **TO-PEG 750**. **(B)** MALDI-ToF mass spectrum of PEG 750 compared to **TO-PEG 750**. The expected and estimated molecular weights (Mw) of **TO-PEG 750** is comparable to each other.

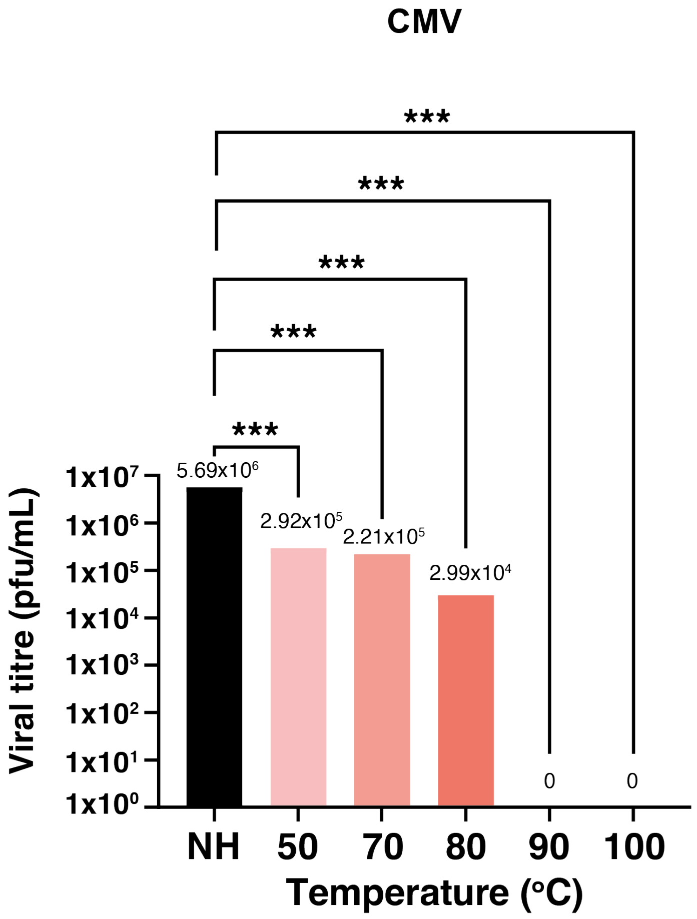

SI Figure 7: Assessing CMV infectivity using standard plaque assay techniques following heating over a range of temperatures (50 °C - 100 °C). Data represents the mean±SD (n=3)

.

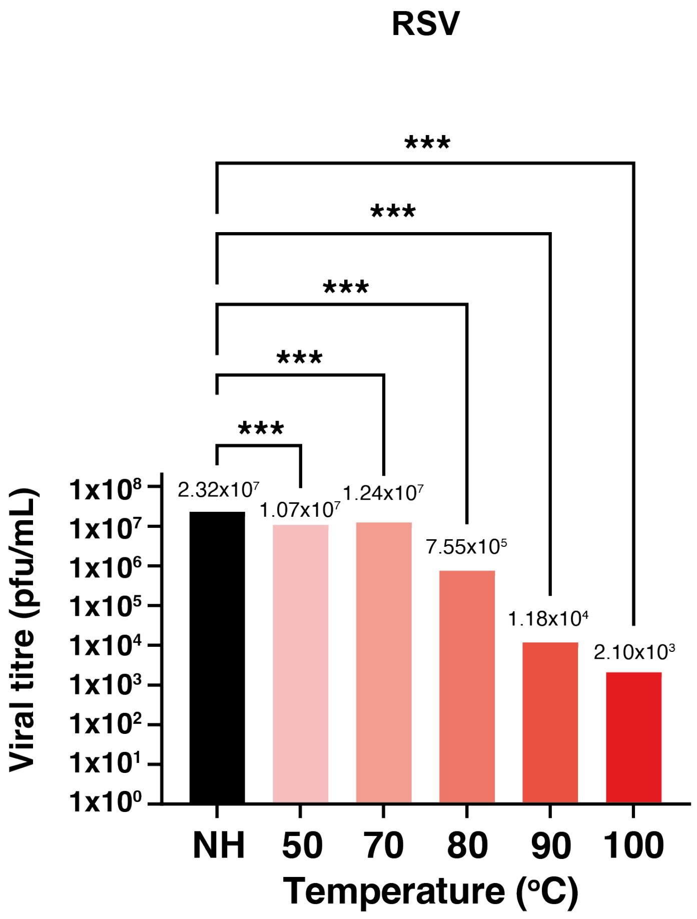

SI Figure 8: Assessing RSV infectivity using standard plaque assay techniques following heating over a range of temperatures (50 °C - 100 °C). Data represents the mean±SD (n=3)

.
